# Lateralization in feeding is food type specific and impacts feeding success in wild birds

**DOI:** 10.1002/ece3.8598

**Published:** 2022-02-07

**Authors:** Karina Karenina, Andrey Giljov

**Affiliations:** ^1^ Department of Vertebrate Zoology Saint Petersburg State University Saint Petersburg Russia

**Keywords:** brain hemisphere dominance, feeding behavior, object discrimination, *Treron phoenicopterus*, visual ecology

## Abstract

Current research suggests that hemispheric lateralization has significant fitness consequences. Foraging, as a basic survival function, is a perfect research model to test the fitness impact of lateralization. However, our understanding of lateralized feeding behavior is based predominantly on laboratory studies, while the evidence from wild animals in natural settings is limited. Here we studied visual lateralization in yellow‐footed green pigeons (*Treron phoenicoptera*) feeding in the wild. We aimed to test whether different types of food objects requiring different searching strategies elicit different eye/hemisphere biases. When feeding on relatively large, uniformly colored food objects (mahua flowers) which can be present or absent in the viewed patch, the majority of pigeons relied mostly on the left eye–right hemisphere. In contrast, when feeding on smaller and more abundant food objects, with color cues signaling its ripeness (sacred figs), right‐eye (left‐hemisphere) preference prevailed. Our results demonstrate that oppositely directed visual biases previously found in different experimental tasks occur in natural feeding situations in the form of lateralized viewing strategies specific for different types of food. The results suggest that pigeons rely on the hemisphere providing more advantages for the consumption of the particular type of food objects, implying the relevance of brain lateralization as a plastic adaptation to ecological demands. We assessed the success of food discrimination and consumption to examine the link between lateralization and cognitive performance. The use of the preferred eye resulted in better discrimination of food items. Discrimination accuracy and feeding efficiency were significantly higher in lateralized individuals. The results showed that visual lateralization impacted pigeons’ feeding success, implicating important fitness benefits associated with lateralization.

## Graphical abstract

The direction of visual lateralization is dependent on the type of food in wild pigeons. The use of the preferred eye resulted in better discrimination of food items. Lateralized individuals showed higher feeding success than non‐lateralized.

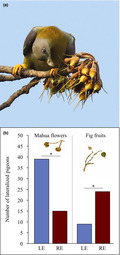

## 1 INTRODUCTION

Lateralized processing of sensory information by the nervous system has been shown to be a property of most bilaterally symmetrical animals (Rogers et al., 2013). Behaviorally, lateralization can be manifested in a form of preferential use or enhanced performance of the sensory organ (e.g., eye, ear, or nostril) on the left or right side of the animal body. The interpretation of such one‐sided biases found in a wide range of behaviors is based on the concept of the asymmetrical neural control of specific cognitive functions like social recognition, spatial memorization, food discrimination, and many others (Rogers, 2017; Siniscalchi, 2017). Recent inroads into the evolutionary origins of the prevalence of lateralization provide compelling evidence for the advantages (and some balancing disadvantages, see, e.g., Chiandetti, 2011; Dadda et al., 2009) of having a lateralized cognition (Corballis, 2020; Frasnelli & Vallortigara, 2018; Vallortigara & Rogers, 2020). The significant associations between fitness traits and lateralization have been repeatedly shown in animal behaviors including key survival functions such as foraging, predator avoidance, and social competition. For example, domestic pigeons showing a higher degree of visual lateralization were more successful in discriminating grain from grit (Güntürkün et al., 2000). In domestic chicks, *Gallus gallus domesticus*, lateralized individuals performed simultaneous food discrimination and predator detection better than their non‐lateralized counterparts (Rogers et al., 2004). In a social context, domestic pigs, *Sus scrofa domesticus*, with stronger lateralization in the orientation towards their opponent have shorter contest duration (i.e., enjoy an advantage in conflict resolution; Camerlink et al., 2018), and starlings with stronger lateralization of social signal processing showed social skill advantages (Cousillas et al., 2020).

Potential benefits of cerebral lateralization are not limited to the enhanced performance of lateralized individuals in the particular tasks in which their lateralized behavior is evident. A higher degree of lateralization has been related to a generally better cognitive ability (Vallortigara & Rogers, 2020). Parrots showing significant foot preference outperformed non‐lateralized parrots in experimental problem‐solving (Magat & Brown, 2009). In fish, individuals showing strong preference in a detour test occupied more favorable positions inside the school and potentially benefited from greater protection from predators (Bisazza & Dadda, 2005). Among invertebrates, larval antlions with significant righting preference showed improved learning abilities as compared to the non‐lateralized individuals (Miler et al., 2017). It is assumed that the enhanced cognitive skills in lateralized individuals may be explained by their improved ability to act directly on many sources of information simultaneously (Rogers, 2000). Furthermore, specialization of one hemisphere on a particular function helps to avoid competition between two hemispheres when that specific function needs to be implemented. This, in turn, may lead to more rapid and efficient reactions to environmental functions (Vallortigara & Rogers, 2005, 2020). However, the association between a strong manifestation of cerebral lateralization and fitness benefits appears to be not that straightforward. For example, in goldbelly topminnows, *Girardinus falcatus*, non‐lateralized fish outperform lateralized ones in some visually guided spatial tasks (Dadda et al., 2009), and in wild gray squirrels, *Sciurus carolinensis*, a negative relationship between the strength of motor lateralization and learning speed was found (Leaver et al., 2020).

The visual system of birds is a well‐established research model to examine the function of cerebral lateralization (Halpern et al., 2005). Behavioral and neurobiological studies demonstrate that birds possess highly advanced cognitive and visual abilities, comparable to those of primates (Lazareva et al., 2020; Soto & Wasserman, 2014). In the avian brain, the optic nerves cross virtually completely, and the input from the left eye is mostly confined to structures of the right hemisphere and vice versa (Rashid & Andrew, 1989; Workman & Andrew, 1986). Therefore, visual lateralization in birds can be easily tested by temporarily occluding one eye with an eye cap. Using this method, chicks were found to discriminate food and non‐food objects significantly better when they rely only on the input from the right eye (the visual importation is processed by the left hemisphere) than when they rely only on the information seen by the left eye and processed by the right hemisphere (Mench & Andrew, 1986). Similarly, domestic pigeons, *Columba livia*, and zebra finches, *Taeniopygia guttata* (Alonso, 1998), performed the food discrimination experimental task more successfully under right‐eye seeing conditions (Güntürkün et al., 2000).

The left and the right brain hemispheres of birds can be tested separately not only by means of monocular occlusion. In many bird species, the eyes are positioned laterally on the sides of their head and the visual fields of the two eyes are largely independent with only a small binocular overlap. As a result, the viewed stimuli cannot be seen binocularly for much of the time and birds adopt independent scanning movements to inspect the environments (Andrew, 1991), for example, during foraging. The preferred side, on which the bird is turning its head to use the monocular visual field, can serve as a behavioral marker to measure visual lateralization unobtrusively. In wild birds, this methodological approach was applied to find that laughing kookaburras, *Dacelo novaeguineae*, predominantly use their left eye to scan for prey at a distance (Rogers, 2002), while black‐winged stilts, *Himantopus himantopus*, show the right‐eye advantage for close‐up food discrimination (Ventolini et al., 2005). In experimental settings, the assessment of birds’ visual preferences without monocular occlusion can be conducted by presenting the bird with multiple food objects regularly scattered on an area in front of it. A preference to peck the grains into the left hemifield when seeing with both eyes was found in both domestic chicks and pigeons (Diekamp et al., 2005).

The substantial evidence from laboratory experiments together with some limited field studies’ results indicate that feeding behavior in birds is characterized by pronounced visual lateralization, and this lateralization is linked to fitness benefits (Güntürkün et al., 2000; Magat & Brown, 2009; Rogers et al., 2004; Ventolini et al., 2005). The results of studies investigating feeding under different circumstances are contrasting (e.g., Ventolini et al., 2005 vs. Diekamp et al., 2005), suggesting that the direction of lateral biases in feeding is context‐dependent in birds. Consequently, the relative roles of the two brain hemispheres seem to vary according to the requirements of the particular feeding situation (reviewed in Rogers & Kaplan, 2019).

It is widely accepted that a better understanding of the functional organization of lateralized brains can be achieved by investigating ecologically valid settings (e.g., Manns, 2021). To date, our understanding of how the complex pattern of lateralized feeding behavior is manifested in wild birds in natural settings is very limited. Do different types of food objects requiring different searching strategies elicit different eye/hemisphere biases? Is lateralization associated with enhanced success in visually guided foraging? In the present study, we tried to shed some light on these questions by means of investigation of feeding situations resembling those in the previous laboratory experiments but occurring naturally in the wild. Preferences for the left or right eye use in feeding on distinct types of food objects were examined unobtrusively in wild yellow‐footed green pigeons, *Treron phoenicoptera*.

To assess visual lateralization in situations characterized by distinct cognitive demands, we observed pigeons feeding on two types of food. The food object characteristics were considered as a valuable variable for the pigeons since laboratory experiments demonstrated their advanced perceptual categorization and discrimination abilities, for example, pigeons can simultaneously attend to four different dimensions of complex visual stimuli (Teng et al., 2015). One type of food studied was larger and more discrete, uniformly colored objects which can be present or absent in the viewed patch. The focus on the food detection resembled the requirements of the experimental task with scattered grains used to test the lateralization of visuospatial attention in birds (Diekamp et al., 2005). Another type of food studied was smaller and more abundant food objects with color cues signaling its ripeness. In this case, the need to distinguish ripe fruits from those not ready to consume resembled the conditions of pebble‐grain test used to study the lateralization of birds’ discrimination abilities (e.g., Alonso, 1998; Güntürkün et al., 2000; Mench & Andrew, 1986). To examine the possible link between lateralization and cognitive performance, besides the visual lateralization during feeding, we also recorded the success of food detection and discrimination. For this purpose, we assessed food discrimination accuracy in lateralized and non‐lateralized pigeons and compared the pecking error rates under left‐eye and right‐eye viewing conditions. The expression of behavioral lateralization in the individual has been linked to foraging efficiency (ingestion rate) in birds and other animals (e.g., Beauchamp, 2013; McGrew & Marchant, 1999; Schnell et al., 2016). Therefore, we compared feeding efficiency between lateralized and non‐lateralized pigeons and between left‐ and right‐eye preferent pigeons.

## 2 MATERIALS AND METHODS

### 2.1 Study sites and subjects

Data on feeding behavior of a largely arboreal fruit‐eating species (Ali & Ripley, 1981), yellow‐footed green pigeon, *Treron phoenicoptera* (henceforth pigeons), were collected at two sites in Madhya Pradesh, India: “Pench” study site and “Kanha” study site, spaced apart from each other at a distance of 134 km. *T*. *phoenicoptera* is a common resident species at both study sites (Chandra et al., 2006; Pasha et al., 2004). Single individuals, pairs, and small flocks of pigeons were observed during their feeding visits to trees. Open spaces between the single trees and almost absent foliage (because of drought) provided good visibility. The trees on which the pigeons were observed were situated in the rural areas bordering the national parks of the same name. The trees were standing alone or in small clusters of several trees with numerous paths around them. These paths were regularly used by local villagers moving around by foot or bicycle. As a result, the resident birds were well habituated to often and non‐threatening encounters with humans. The presence of researchers near the trees did not elicit visible disturbance to the birds feeding on the trees.

At each study site, pigeons were observed on six different trees, three of each of two species studied (see details in the subsection 2.2). Pigeons from one flock (arriving and leaving together) were traced individually based on their location on the tree that was possible thanks to the small sizes of the flocks (mean = 6 individuals, SD = 3) and distancing maintained by pigeons feeding together. The data sampling procedure and the abundance of the species in the study areas (Chandra et al., 2006; Pasha et al., 2004) minimized the probability of repeated observations of the same individuals. We always observed pigeons coming to the particular tree from one direction and leaving it in another direction. Thus, pigeons likely moved from one tree to another without repeated visits during the same day that corresponds with the pattern of foraging movements typical for fruit‐eating birds (Snow & Snow, 2010; Wheelwright, 1991). To further minimize the probability of repeated observations of the same individuals, we conducted observations at each study tree during a single continuous session (one day – one tree).

To test the potential impact of repeated observations of the same individuals we (a) compared the data between study trees and (b) compared the data on birds from the same flock (definitely different individuals) and from a synthetic flock (randomly assigned individuals from different flocks; see Data analysis for details). The data collected in Kanha and Pench were evidently from different individuals because of the distance between the two study sites.

### 2.2 Feeding conditions

At each study site, pigeons were observed feeding on two species of trees: mahua tree, *Madhuca longifolia*, and sacred fig, *Ficus religiosa*. The distance between six study trees at one study site (three trees of each species) ranged between 320 and 1307 m.

Mahua is a medium to large deciduous tree found in many parts of India and belonging to the family Sapotaceae. Mahua tree flowers are characterized by thick fleshy corollas which is the adaptation for pollination by fruit bats (Nathan et al., 2009). Flowers are well exposed attracting besides bats many frugivorous birds. Pigeons were observed feeding on flower corollas about 21 mm long and 15 mm wide clustered around the branch tips (Patel et al., 2011). Mahua flowers are growing in discrete inflorescences consisting of multiple flowers with thick corolla (picked by birds) or lacking it (immature, decayed, or eaten).

**FIGURE 1:**
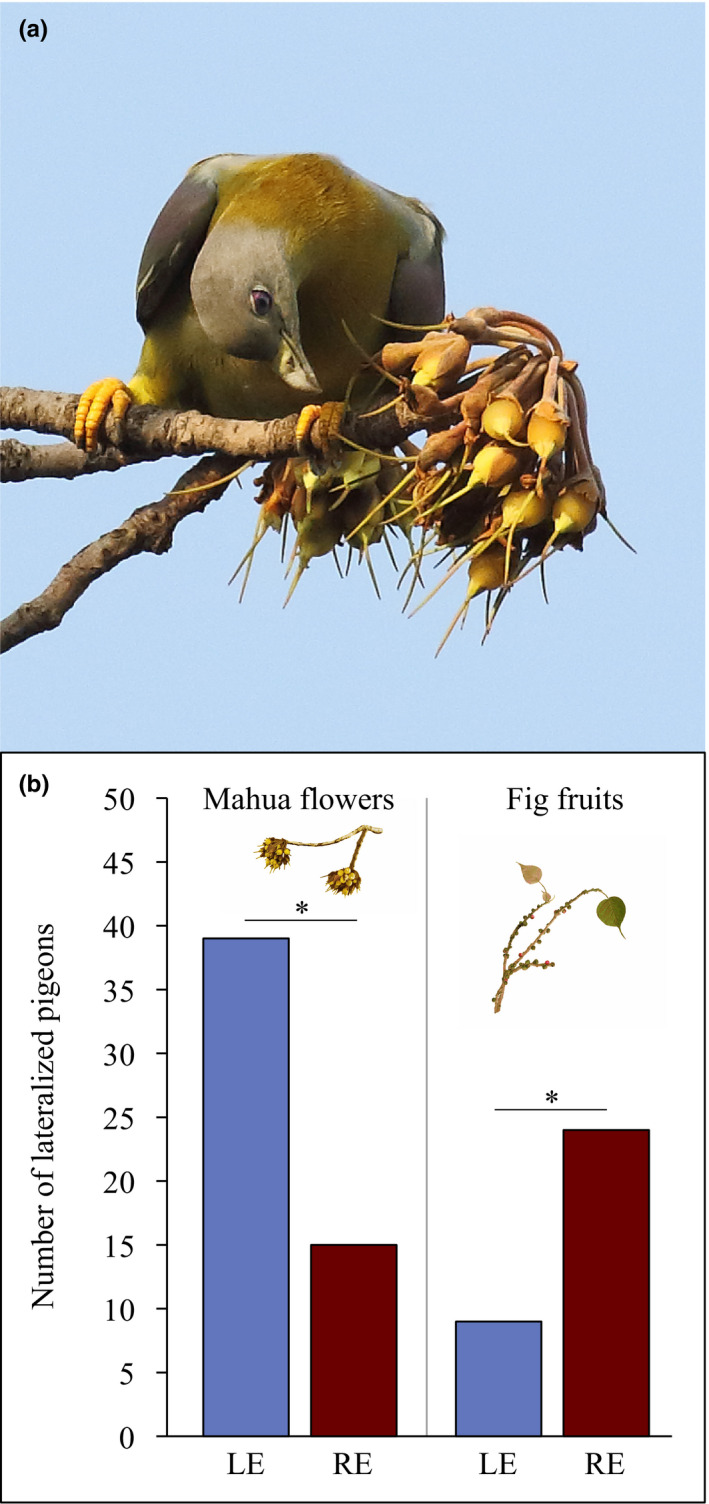
Visual lateralization in feeding on different types of food items. (a) A yellow‐footed green pigeon is about to peck a mahua tree flower after inspecting a patch with the left eye. (b) When feeding on mahua flowers (left), the significant majority of lateralized individuals showed the preference to view the patch with the left eye (LE) prior to pecking. When feeding on sacred fig fruits (right), the majority of lateralized pigeons preferred to use the right eye (RE). *p < .05

Sacred fig, also known as peepal, is a large deciduous tree native to the Indian subcontinent and Indochina that belongs to Moraceae family. The fruits are small figs 1–1.5 cm in diameter scattered along the distal parts of the branches. Pigeons searched for the blackish purple ripe fruits, contrasting with the green immature figs.

The general behavioral pattern observed in feeding yellow‐footed green pigeons can be described as follows. The single bird, a couple, or a small flock landed on the top of the tree and looked around for some time. After that, the birds fluttered down on the chosen branch to start foraging. In flocks, individual birds usually occupied different parts of the tree and steadily kept a distance from each other while feeding. When looking for food, pigeons followed the pattern of visual search typical for birds with primarily monocular vision (Andrew & Dharmaretnam, 1993). After approaching the branch tip where most flowers/fruits were clustered, the birds examined the patch monocularly (indicated by lateral head position; Figure 1a). Monocular fixation was followed by either the further move to another patch (if no food was detected) or orienting the head towards a potential food item and making a peck under control of binocular vision.

### 2.3 Data collection

The feeding behavior was recorded by the two observers with binoculars and voice recorders, tracing individual pigeons from their first landing on the tree till leaving. If more than two pigeons visited the focal tree simultaneously, individuals for observation were chosen at random and other pigeons were ignored. Observations and voice recordings were chosen over video recordings as they allowed the researcher to move freely and focus visual attention on a focal pigeon moving from branch to branch in search of food.

In total, the pigeons visiting each study tree were observed during continuous 4–5 h‐long sessions which started at dawn. Two subsamples of data (50 feeding visits of individual pigeons per each type of food object at the “Pench” study site) were scored by both raters (KK and AG) independently at the beginning of the study. The degree of agreement between the two raters was quantified by kappa. In determining the eye used for the monocular inspection, the inter‐rater agreement was 90% (kappa = 0.84, SE of kappa = 0.07) for the mahua flowers and 88% (Kappa = 0.82, SE of kappa = 0.07) for the sacred fig fruits. Since this agreement between the two raters corresponds to the “almost perfect agreement” level (Landis & Koch, 1977), we further made the observations simultaneously but on different individuals.

For each individual pigeon, we recorded every peck during the feeding visit. Based on the head position, the eye (left/right) used for monocular inspection of the patch prior to peck was recorded. If no monocular inspection was evident before the peck, it was recorded as non‐lateral. Whether or not the peck was successful was assessed based on the presence of swallowing head movement after the peck. From the voice recording, we subsequently scored the time from landing to the first peck of a flower/fruit and the total time of the feeding visit. The time when the pigeon “froze” for more than 5 s without displaying food searching behavior was excluded from the total feeding time. In the rare cases when the feeding was interrupted by social interactions with another individual, the observation was ceased and this individual was further excluded from the analysis to minimize the potential effect of social factors on lateralization and feeding success scores.

### 2.4 Data analysis

Only the pigeons with at least 15 monocular inspections were included in the analysis. A number of left‐ and right‐eye inspections were compared with a binomial *z* test to classify each individual as having left/right eye preference or being non‐lateralized. To test population bias, lateralization index (LI) was calculated for each individual using the formula LI = (L− R)/(L + R), where L and R are the number of the left‐ or right‐eye inspections. LI scores range on a continuum from −1.0 to 1.0, with positive values indicating the left‐side bias and negative values indicating the right‐side bias. The absolute values of the LI (ABS‐LI) were used to assess the strength of preference. Population‐level lateralization based on LI scores was tested with a one‐sample Wilcoxon signed rank test, while the impact of the type of food objects on the strength of preference (ABS‐LI) was explored using a Mann‐Whitney test.

We analyzed the lateralization impact on feeding accuracy and efficiency based on the frequency of pecking errors (pecks not resulted in food consumption) and ingestion rate (food items consumed per minute), respectively. The frequency of pecking errors was compared between lateralized and non‐lateralized pigeons (Mann–Whitney test). Further, in lateralized individuals, we assessed the influence of the eye used for inspection of the patch on the accuracy of the peck by comparing the proportion of the errors made under left‐ and right‐eye viewing conditions (Wilcoxon matched‐pairs signed rank test). For this analysis, we used a subsample of lateralized pigeons with six pecking errors per individual. The birds which made fewer errors were excluded. In birds with more than six errors, only the first six were used for a balanced design (Tables S1 and S2). For the analysis of feeding efficiency, we scored the ingestion rate of each individual (Tables S1 and S2). The total time the pigeon spent feeding and the total number of successful pecks made (both lateral and non‐lateral) were used. The ingestion rates were compared between lateralized and non‐lateralized pigeons and between the left‐ and right‐lateralized pigeons with a Mann‐Whitney test.

To investigate the influence of potential sampling of the same individual within different flocks, we applied a Monte Carlo simulation approach and generate synthetic flock datasets (Table S7). A real flock (all individuals are different) was compared with ten simulated flocks of the same size (Kruskal–Wallis test with the real flock as a control). Each simulated flock comprised randomly assigned individuals from other flocks (potentially, some individuals are actually the same).

The data from two study sites were compared to test the consistency of lateralization for each type of food with a multinomial regression with individual preferences (L, R, N, based on binomial *z* test) as a dependent variable and study sites as factors as well as with Mann‐Whitney test on LI scores. For both mahua flowers and fig fruits, we used LI scores to test relationships between lateralization in pigeons and (a) a particular tree on which feeding birds were observed, and (b) the flock, the pigeon belonged to using linear regression analyses (LI vs. particular tree/flock) and Kruskal–Wallis tests (based on LIs).

In the case of feeding on sacred fig fruits, almost every peck was followed by swallowing movement. Therefore, only the data on feeding on mahua tree flowers were used for the analysis of feeding accuracy. The percentage of pecking errors (pecks not followed by swallowing, i.e., not resulted in food consumption) to total lateral peck was compared between lateralized and non‐lateralized individuals (Mann–Whitney test).

All analyses were two‐tailed with α set at 0.05.

## 3 RESULTS

### 3.1 Pigeons show object‐specific visual lateralization in feeding

In total, the consumption of 2480 mahua flowers by yellow‐footed green pigeons was analyzed (median = 32, 95% CI: 29–34). For 1565 flowers monocular inspection prior to pecking was recorded (median = 20, 95% CI: 18–21). We recorded 15 or more monocular inspections of mahua tree flowers for 74 pigeons (44 at the “Pench” study site and 30 at the “Kanha” study site).

For the sacred fig, we recorded consumption of 1778 fruits (median = 43, 95% CI: 33–51), and for 930 fruits monocular inspection prior to pecking was observed (median = 20, 95% CI: 17–24). For a total of 43 pigeons 15 or more monocular inspections was recorded (25 at the “Pench” site and 18 at the “Kanha” site).

Time from landing on a tree to the first peck (feeding latency) was higher during feeding on sacred fig fruits (mean 15.0 ± 1 s) than during feeding on mahua tree flowers (mean 8.7 ± 0.5 s; Mann–Whitney *U* = 688, *p* < .001; Tables S1 and S2).

The significant majority of pigeons displayed individual preferences for one eye when inspecting both mahua flowers (73%, 54 out of 74, binomial *z* = 3.84, *p* < .001) and fig fruits (77%, 33 out of 43, binomial *z* = 3.35, *p* < .001; Tables S1 and S2). When feeding on mahua flowers, more lateralized individuals showed a preference for the left eye (72%, 39 out of 54, binomial *z* = 3.13б, *p* = .001). In contrast, when feeding on fig fruits, the majority of lateralized pigeons had a right‐eye preference (73%, 24 out of 33, binomial *z* = 2.44, *p* = .014; Figure 1b). The analysis of population‐level lateralization based on LI scores confirmed the general left‐eye preference for mahua flowers (one‐sample Wilcoxon signed rank test, *W* = −1170, *p* = .001) and the right‐eye preference for fig fruits (*W* = 439, *p* = .007). While the direction of lateralization was different for mahua flowers and fig fruits, the strength of lateralization (based on Abs‐LI) did not differ between two types of food objects (Mann–Whitney *U* = 1295, *p* = .094).

### 3.2 Visual preferences impact feeding accuracy and efficiency

To test the impact of lateralization on feeding accuracy during feeding on mahua flowers, the frequency of pecking errors was compared between pigeons showing significant visual preferences and non‐lateralized pigeons. The analysis showed that in non‐lateralized individuals, the accuracy was significantly lower (66% of successful pecks) as compared to lateralized individuals (74%, Mann–Whitney *U* = 174.5, *p* < .001; Tables S1 and S2). No pecking errors were recorded for feeding on sacred fig fruits.

In lateralized individuals, we further compared the error rate was under left‐ and right‐eye viewing conditions during feeding on mahua flowers. The proportion of pecks not resulted in food consumption was higher when the pigeons used their non‐preferred eye. The individuals with the right‐eye preference were more likely to make pecking errors when they used their left eye for food inspection prior to the peck (Wilcoxon matched‐pairs signed rank test, *W* = 62, *p* = .011), and vice versa; the left‐lateralized pigeons were more likely to make an error after the right‐eye inspection (*W* = −252, *p* = .002).

To assess the effect of lateralization on individuals’ feeding efficiency, we compared the ingestion rate between lateralized and non‐lateralized pigeons and between right‐lateralized and left‐lateralized pigeons. The efficiency of feeding was significantly lower in non‐lateralized individuals for both types of food (Mann‐Whitney test, mahua flowers: *U* = 175.5, *p* < .001; fig fruits: *U* = 89.5, *p* = .029). When feeding on fig fruits, individuals with the right‐eye preference were significantly more efficient than individuals with the left‐eye preference (*U* = 46.0, *p* = .013). At the same time, no difference in feeding efficiency was found between left‐ and right‐lateralized pigeons during feeding on mahua flowers (*U* = 231, *p* = .240). In addition, we found that time between landing on a tree to the first peck was shorter in lateralized pigeons than in non‐lateralized for both types of food objects (Mann–Whitney test, mahua flowers: *U* = 235, *p* < .001; fig fruits: *U* = 34, *p* < .001).

### 3.3 Lateralization is not influenced by the study site, particular tree or flock

The lateralization in food object inspection (based on LI scores) did not differ between two study sites either for mahua flowers (Mann–Whitney *U* = 594.5, *p* = .475) or fig fruits (*U* = 220.0, *p* = .908). A multinomial regression analysis on the distribution of left, right, and non‐lateralized pigeons (based on individual *z* scores) also failed to reveal the influence of the study site (*p* > .05, Table S3).

Results of linear regression analysis did not show any significant relationships between lateralization in pigeons (based on LI) and a particular tree on which feeding birds were observed for both mahua flowers and fig fruits (*p* > .05, Table S4). Kruskal‐Wallis tests failed to reveal the difference in lateralization in all but one comparison of LI scores sampled from pigeons feeding on different trees (*p* > .05, Table S5).

No influence of the feeding flock, the pigeon belonged to, was revealed either for mahua flowers or for fig fruits (linear regression, *p* > .05, Table S6). To investigate the effect of potential sampling of the same individuals within different flocks, we compared LI scores between a real flock (all individuals are different) and ten simulated flocks of the same size comprising random individuals from other flocks (for details, see the supplementary methods section). Kruskal–Wallis tests did not show any significant difference (*p* > .05, Table S7), suggesting no influence of potential repeated sampling on the lateralization results.

## 4 DISCUSSION

### 4.1 Lateralization in feeding

The results demonstrate lateralization of visual inspection of food objects in yellow‐footed green pigeons in the wild. Regardless of the type of food, the majority of individuals displayed preferences for one eye when viewing the patch of flowers/fruits just before pecking and consuming a food item. The direction of lateralization at the population level differed according to the two types of food consumed. An overall preference for the left eye was found in feeding on flowers of mahua tree. In contrast, when feeding on sacred fig fruits, pigeons showed a population preference for the right eye. The most plausible explanation of these differences is the impact of the food objects’ properties on the cognitive demands involved in feeding and, consequently, on the division of the hemispheric roles.

Pigeons can simultaneously attend to four different dimensions of complex visual stimuli (Teng et al., 2015). That implies that the visual characteristics of the food objects is a valuable variable for the cognitive processing involved in food search and consumption. The two types of food object studied have distinct visual properties. Mahua tree flowers grow is bundles and are relatively large and discrete food objects, with no significant color differences. The mature flowers with thick fleshy corollas consumed by birds can be present or absent in the viewed patch. Smaller food objects, sacred fig fruits, are more numerous scattered within a single patch and have color cues signaling its ripeness, with ripe fruits preferred by birds. Functionally distinctive cognitive processes, for example, attention to different dimensions of the visual stimuli, required for the consumption of the two types of food objects may result in the greater involvement of different hemispheres. That is, different processing modes involved in the consumption of the two types of food may drive differential lateralized inspection in green pigeons.

The observed behavioral differences further support the idea that feeding on mahua flowers and fig fruits are distinctive tasks for pigeons. The feeding latency (time from landing on a tree to the first peck) was significantly higher in the case of sacred fig than in the case of mahua tree. That is, pigeons needed more time to look for the ripe fruits and choose the most promising patch to start feeding. In addition, when pigeons were feeding on fig fruits, almost every peck was followed by swallowing, implying a very low error rate in choosing a food item suitable for consumption. Feeding on mahua flowers, in contrast, was associated with a noticeable portion of unsuccessful pecks (not followed by the food item consumption), indicating a higher error rate and, potentially, more difficulties in making the correct choice of the food item. This result implies that the two types of food objects studied here require different types of analysis to be involved in feeding.

Considering feeding on two types of food as two distinctive cognitive tasks, we can further try to explain the directions of the revealed preferences. A large amount of evidence on vertebrates indicates that the brain hemispheres use distinctive cognitive strategies, with the right hemisphere relying mostly on more systematic and spatially focused analysis, and the left hemisphere takes responsibility for categorical distinctions and the selection of targeted stimulus from among alternatives (e.g., Halpern et al., 2005; Vauclair et al., 2006). When feeding on a mahua tree, pigeons are looking for the presence of edible flowers with thick fleshy corollas among immature or decayed flowers that requires systematic searching with the attention to spatial relations between objects. As these are known properties of the right hemisphere, the left‐eye preference found in pigeons feeding on mahua flowers corresponds to the general pattern of hemispheric functions. In experimental settings, domestic chicks and domestic pigeons preferentially peck the grains into the left visual hemifield (Diekamp et al., 2005), implicating better detection of food items by the right hemisphere. The left eye–right hemisphere preference has also been found in kookaburras which, much like pigeons in our study, were observed unobtrusively in the wild (Rogers, 2002). The author suggests that the preference to scan for prey with the left eye indicates attention to the spatial location of potential food object. In green pigeons too, the left eye preference may be explained by the primary involvement of spatial analysis in the detection of suitable mahua flowers.

The right‐eye preference for feeding on sacred fig fruits corresponds to the known properties of the left brain hemisphere. Choosing a ripe fig to consume, a pigeon faces many alternative targets placed close together and belonging to different categories of ripeness. The ability of the left hemisphere to generate categorical distinctions (Manns et al., 2021; Vauclair et al., 2006) most likely favors the use of the right eye for feeding on figs. Previously, the right eye (left hemisphere) advantage for discrimination of food and non‐food objects has been found in several bird species (Alonso, 1998; Güntürkün et al., 2000; Mench & Andrew, 1986). In the experimental task, birds performed better in the selection of food grains from grains of similar size under right‐eye seeing conditions than under left‐eye seeing conditions. In addition, the left brain hemisphere plays the dominant role in color discrimination in pigeons (Verhaal et al., 2012), implying that discrimination of ripe figs by color may trigger left‐hemispheric processing and preferential right‐eye use. Thus, the specific dimensions of the potential food object are better recognized by the left hemisphere. It is conceivable that feeding on a fig tree, pigeons rely predominantly on the right eye because the left hemisphere provides advantages in the discrimination of ripe fruits.

To conclude, our results suggest that specific characteristics of the food objects may trigger different hemispheric dominance, and green pigeons rely on the hemisphere providing more advantages for the consumption of the particular type of food. In other words, pigeons adopt different viewing strategies depending on the type of cognitive task involved in the particular feeding situation. Our results, corroborating previous research (e.g., Rogers & Kaplan, 2019), do not indicate the specialization of one hemisphere on the control of feeding behavior but demonstrate the flexibility of hemispheric dominance as a plastic adaptation to ecological demands. The flexibility in adopting hemispheric‐specific processing strategies depending on the feeding context may be crucial for understanding fitness advantages associated with lateralization. Somewhat similar results indicating specialization of lateralized feeding strategies have been previously reported for whales. The direction of individuals’ lateralization during lunge feeding in blue whales, *Balaenoptera musculus*, depended on where and how the behavior was performed. The larger rolls during lunging targeting small, less dense krill patches near the water's surface were more likely to be left‐lateralized than the smaller rolls during deep lunges (Friedlaender et al., 2017). Authors suggest that distinct lateralized feeding strategies may enhance foraging efficiency in environments with heterogeneous prey distributions. Thus, the specialization of lateralized behavior for different feeding circumstances may be a widespread phenomenon, indicating the importance of considering feeding context in lateralization research.

### 4.2 The link between lateralization and feeding success

The results of the study demonstrated the association between visual lateralization and foraging success in two ways. First, we compared the number of pecking errors (pecks not resulted in food consumption) when feeding on mahua flowers in lateralized and non‐lateralized pigeons and found the impact of lateralization on food discrimination accuracy. Pigeons with significant visual preferences were more successful in pecking food items suitable for consumption. This result suggests that in pigeons, being lateralized improves food discrimination accuracy and, consequently, enhances feeding success and confers fitness benefits. Furthermore, we found that feeding accuracy was higher when the individuals with the right‐eye preference used their right eye (and the individuals with the left‐eye preference used their left eye) to view the food prior to pecking. Thus, regardless of the direction of preference, the use of the preferred eye provides better discrimination of food items, demonstrating a background of the prevalence of individual‐level lateralization in the population.

Improved accuracy in lateralized green pigeons feeding in the wild corroborates previous laboratory findings on domestic pigeons. In the experiment including monocular occlusion, pigeons with stronger visual lateralization were more successful in food object discrimination than their weakly lateralized counterparts (Güntürkün et al., 2000). Beyond the foraging context, the discrimination accuracy has been investigated in the recognition and rejection of avian brood parasitic eggs. American robins, *Turdus migratorius*, egg‐rejecter hosts, which were more lateralized in the inspection of clutch showed higher rates of model eggs rejection (Scharf et al., 2019). In line with this, the electrophysiological results on starling, *Sturnus vulgaris*, revealed a positive correlation between lateralized social signal processing and individual social integration (Cousillas et al., 2020). Thus, the cumulative results on birds illustrate one of the most obvious advantages of hemispheric lateralization—the lateralized implementation of the particular task is associated with better performance. This is assumed to be underpinned by decreased redundancy of neural operations, avoidance of delays resulting from interhemispheric conflict, and improved parallel processing (e.g., Rogers, 2000; Vallortigara & Rogers, 2005, 2020). Results on wild birds demonstrate that this advantage is significant not only in the controlled experimental conditions but also in more ecologically valid settings.

The second analysis which indicated the link between lateralization and feeding success was based on the comparison of feeding efficiency reflected in ingestion rate (food items consumed per minute) between lateralized and non‐lateralized pigeons. Faster consumption can be especially advantageous in the case of foraging on a fruiting (flowering) tree when the food is available only for a limited time, and there are numerous hetero‐ and conspecific competitors around (Snow & Snow, 2010). For both mahua flowers and sacred fig fruits, the feeding efficiency of individuals was significantly higher in lateralized pigeons, implicating important fitness benefits associated with lateralization. Besides faster consumption, lateralized pigeons started to feed sooner after landing on a tree than their non‐lateralized counterparts. This suggests a higher speed of decision making in lateralized pigeons and highlights general differences in the behavioral patterns of individuals with different levels of lateralization. Our results support the growing amount of evidence for a positive association between the strength of lateralization and cognitive performance. Some limited but diverse studies have demonstrated that more strongly lateralized individuals tend to cognitively outperform weakly lateralized individuals (e.g., in invertebrates: Miler et al., 2017; fish: Bisazza & Brown, 2011; birds: Magat & Brown, 2009; and humans: Nettle, 2003).

If lateralized green pigeons outperform non‐lateralized ones in feeding, why then are the non‐lateralized individuals preserved in the population? While acknowledging the growing amount of evidence for the advantages of lateralized behavioral responses, it is important to note that lateralization has some balancing disadvantages (reviewed in Rogers, 2002 and Frasnelli & Vallortigara, 2018). A number of previous studies report the degree of lateralization to be negatively correlated with success in performing particular tasks. For example, in goldbelly topminnows, non‐lateralized fish outperform lateralized ones in some visually guided spatial tasks (Dadda et al., 2009). In antlion larvae, *Myrmeleon bore*, weakly lateralized individuals detect and capture prey more quickly than the individuals with stronger lateralization (Miler et al., 2018). Thus, the optimal degree of lateralization that an individual should have may depend on the task and functional context. That is, the manifestation of benefits associated with lateralization is more complex than a simple principle “the more lateralized, the better.” In green pigeons, individuals showing no lateralization in feeding, and, consequently, having lower feeding success may potentially outperform lateralized individuals in other tasks. In other words, the non‐lateralized pigeons may persist in the population because the degree of lateralization that is beneficial in one task may not be beneficial in another.

The influence of the direction of lateralization on feeding efficiency was revealed for feeding on fig fruits. The individuals with the right‐eye preference fed faster than the individuals with the left‐eye preference. That is, the prevalent type of lateralization in the population (right eye preference) was associated with greater feeding success. From an evolutionary standpoint, this result may illustrate the simple background for the unequal numbers of left‐ and right‐lateralized individuals in the population. As the individuals with the right‐eye preference enjoy fitness benefits from higher feeding efficiency, they have a greater chance of survival and, consequently, prevail in the population.

For mahua flowers, no significant difference in feeding efficiency was found between the individuals lateralized in the opposite directions. This result indicates that while being lateralized increases consumption rate, the particular direction of the lateralization does not further improve it. Why then do the pigeons with the left‐eye preference for feeding on mahua flowers prevail in the population? It is conceivable, that while the greater involvement of the right hemisphere doesn't result in faster food consumption, it confers other important advantages to the individuals. Some specific functions of the right hemisphere, for example, the superiority in the decisions based on a memory‐based exemplar strategy (Halpern et al., 2005) or processing of configurational information (Vauclair et al., 2006), may help pigeons to detect better food items rather to consume them faster. For example, consumption of more mature, energetically profitable mahua flowers may confer fitness advantages to pigeons making the left‐eye viewing strategy preferable without its influence on the speed of feeding.

To conclude, our results demonstrate how different visual lateralizations, resembling those previously found in laboratory experiments, occur in real‐life feeding situations as distinct strategies of lateralized viewing specific for different types of food. The revealed impact of visual preferences on feeding success provides further evidence that behavioral lateralization has important fitness consequences for animals in their natural environments.

## CONFLICT OF INTEREST

We have no competing interests.

## AUTHOR CONTRIBUTIONS

**Karina Karenina:** Conceptualization (equal); Data curation (equal); Formal analysis (equal); Funding acquisition (equal); Investigation (equal); Methodology (equal); Project administration (equal); Resources (equal); Supervision (equal); Validation (equal); Visualization (equal); Writing – original draft (equal); Writing – review & editing (equal). **Andrey Giljov:** Conceptualization (equal); Data curation (equal); Formal analysis (equal); Funding acquisition (equal); Investigation (equal); Methodology (equal); Project administration (equal); Resources (equal); Supervision (equal); Validation (equal); Visualization (equal); Writing – original draft (equal); Writing – review & editing (equal).

### OPEN RESEARCH BADGES

This article has earned an Open Data Badge for making publicly available the digitally‐shareable data necessary to reproduce the reported results. The data is available at https://doi.org/10.6084/m9.figshare.16574840.

## Supporting information

Tables S1–S7

## Data Availability

Data are available in the figshare repository, https://doi.org/10.6084/m9.figshare.16574840.
