# Supplementary material for: Lateralization in feeding is food type specific and impacts feeding success in wild birds
Source: Ecol Evol. 2022 Feb 7;12(2):e8598. doi: 10.1002/ece3.8598 (PMC8820115; doi:10.1002/ece3.8598)
Supplement: Supplementary file 1 — Tables S1–S7 [file ECE3-12-e8598-s001.pdf]

Supporting Information

Lateralization in feeding is food type specific and impacts feeding success in wild birds

Supplementary Tables

Table S1 Individual data on feeding on mahua tree, *Madhuca longifolia* flowers

**Individual**	**Time from landing to first peck, s**	**Total flowers ingested**	**Successful lateral pecks**		**z**	**p**	**pref**	**LI**	**Lateral pecking errors**		**Successful to total lateral pecks, %**	**Total feeding time, s**	**Igestion rate, flowers/ min**	**Tree**	**Flock**
			**R**	**L**					**R**	**L**					
'Pench' stydy site															
1	2	26	13	2	2.58	0.007	R	0.73	1	4	75	303	5.15	MTP1	MFP1
2	10	34	1	18	-3.67	<0.001	L	-0.89	6	1	73	433	4.71	MTP1	MFP1
3	14	30	13	6	1.38	0.167	N	0.37	2	2	83	481	3.74	MTP1	MFP1
4	5	30	8	11	-0.46	0.648	N	-0.16	5	6	63	506	3.56	MTP1	MFP2
5	11	32	5	15	-2.01	0.041	L	-0.50	5	2	74	498	3.86	MTP1	MFP2
6	12	37	16	5	2.18	0.027	R	0.52	3	5	72	531	4.18	MTP1	MFP2
7	5	26	2	15	2.91	0.002	L	-0.76	5	0	77	404	3.86	MTP1	MFP2
8	14	33	7	14	-1.31	0.189	N	-0.33	6	3	70	553	3.58	MTP1	MFP3
9	7	34	5	15	-2.01	0.041	L	-0.50	5	3	71	503	4.06	MTP1	MFP4
10	12	35	0	21	-4.36	<0.001	L	-1.00	7	2	70	517	4.06	MTP1	MFP4
11	5	29	3	13	-2.25	0.021	L	-0.63	5	1	73	380	4.58	MTP1	MFP5
12	7	26	12	3	2.07	0.035	R	0.60	1	0	94	413	3.78	MTP1	MFP5
13	12	37	12	13	0	1	N	-0.04	6	5	69	514	4.32	MTP1	MFP5
14	3	34	17	3	2.91	0.003	R	0.70	2	7	69	490	4.16	MTP1	MFP5
15	8	40	7	19	-2.16	0.029	L	-0.46	2	5	79	591	4.06	MTP1	MFP5
16	28	46	8	22	-2.37	0.016	L	-0.47	8	3	73	618	4.47	MTP2	MFP6
17	13	52	9	24	-2.44	0.014	L	-0.45	8	6	70	679	4.59	MTP2	MFP6
18	4	28	1	14	-3.10	<0.001	L	-0.87	5	0	75	400	4.20	MTP2	MFP7
19	13	24	4	11	-1.55	0.118	N	-0.47	2	4	71	392	3.67	MTP2	MFP7
20	5	34	3	18	-3.06	0.001	L	-0.71	6	1	75	515	3.96	MTP2	MFP7
21	6	28	15	3	2.59	0.008	R	0.67	1	5	75	423	3.97	MTP2	MFP7
22	12	33	9	11	-0.22	0.824	N	-0.10	4	6	67	539	3.67	MTP2	MFP7
23	7	33	2	17	-3.21	<0.001	L	-0.79	2	6	70	440	4.50	MTP2	MFP7
24	8	37	13	8	0.87	0.383	N	0.24	6	4	68	587	3.78	MTP3	MFP8
25	5	43	6	21	-2.69	0.006	L	-0.56	2	7	75	618	4.17	MTP3	MFP8
26	2	25	0	16	-3.75	<0.001	L	-1.00	0	9	64	401	3.74	MTP3	MFP8
27	6	29	16	4	2.46	0.012	R	0.60	1	6	74	438	3.97	MTP3	MFP8
28	14	31	6	15	-1.75	0.078	N	-0.43	3	7	68	524	3.55	MTP3	MFP8
29	10	50	8	24	-2.65	0.007	L	-0.50	4	8	73	631	4.75	MTP3	MFP8
30	4	43	7	22	-2.60	0.008	L	-0.52	8	2	74	514	5.02	MTP3	MFP8
31	10	45	11	20	1.44	0.150	N	-0.29	11	4	67	649	4.16	MTP3	MFP8
32	7	45	2	25	-4.23	<0.001	L	-0.85	9	3	69	582	4.64	MTP3	MFP9
33	4	27	0	16	-3.75	<0.001	L	-1.00	6	0	73	416	3.89	MTP3	MFP9
34	13	23	2	13	-2.58	0.007	L	-0.73	5	2	68	337	4.09	MTP3	MFP9
35	4	32	14	4	2.12	0.031	R	0.56	1	5	75	450	4.27	MTP3	MFP9
36	3	48	7	24	-2.87	0.003	L	-0.55	0	0	100	616	4.68	MTP3	MFP10
37	9	46	5	27	-3.71	<0.001	L	-0.69	8	0	80	649	4.25	MTP3	MFP10

38	14	32	3	14	-2.43	0.013	L	-0.65	5	1	74	431	4.45	MTP3	MFP10
39	7	27	9	6	0.52	0.607	N	0.20	4	4	65	447	3.62	MTP3	MFP10
40	11	40	12	15	-0.38	0.701	N	-0.11	5	9	66	610	3.93	MTP3	MFP10
41	6	24	13	2	2.58	0.007	R	0.73	1	5	71	354	4.07	MTP3	MFP10
42	10	37	5	19	-2.65	0.007	L	-0.58	7	2	73	566	3.92	MTP3	MFP11
43	5	28	0	18	-4.01	<0.001	L	-1.00	6	0	75	365	4.60	MTP3	MFP11
44	12	25	5	10	-1.03	0.302	N	-0.33	4	3	68	406	3.69	MTP3	MFP11
'Kanha' study site															
1	7	32	19	11	1.28	0.200	N	-0.50	5	6	73	537	3.58	MTK1	MFK1
2	5	23	12	3	2.07	0.035	R	0.60	1	4	75	388	3.56	MTK1	MFK1
3	9	52	24	10	2.23	0.024	R	0.41	2	4	85	411	7.59	MTK1	MFK1
4	8	33	14	8	1.07	0.286	N	0.27	9	4	63	587	3.37	MTK1	MFK2
5	10	35	16	5	2.18	0.027	R	0.52	7	1	72	515	4.08	MTK1	MFK2
6	6	30	13	8	0.87	0.383	N	0.24	6	11	55	584	3.08	MTK1	MFK2
7	1	31	17	3	2.46	0.012	R	0.70	2	5	74	503	3.70	MTK1	MFK3
8	7	26	16	0	3.75	<0.001	R	1.00	3	8	59	388	4.02	MTK1	MFK3
9	12	29	3	16	-2.75	0.004	L	-0.68	0	0	100	505	3.45	MTK1	MFK3
10	15	24	4	11	-1.55	0.118	N	-0.47	11	3	52	404	3.56	MTK1	MFK3
11	7	25	3	13	-2.25	0.021	L	-0.63	2	4	73	416	3.61	MTK2	MFK4
12	5	27	2	15	-2.91	0.002	L	-0.76	5	1	74	404	4.01	MTK2	MFK4
13	3	23	0	15	-3.61	<0.001	L	-1.00	0	5	75	403	3.42	MTK2	MFK5
14	14	42	5	15	-2.01	0.041	L	-0.50	10	3	61	612	4.12	MTK2	MFK6
15	8	23	4	11	-1.55	0.118	N	-0.47	5	5	60	631	2.19	MTK2	MFK6
16	6	40	10	19	-1.49	0.136	N	-0.31	15	8	56	685	3.50	MTK2	MFK6
17	10	24	3	12	-2.07	0.035	L	-0.60	5	0	75	399	3.61	MTK2	MFK6
18	10	26	4	12	-1.75	0.077	N	0.60	7	3	62	406	3.84	MTK3	MFK7
19	12	51	5	23	-3.21	<0.001	L	-0.64	9	3	70	640	4.78	MTK3	MFK7
20	7	28	4	14	-2.12	0.031	L	-0.56	6	7	58	410	4.10	MTK3	MFK7
21	6	27	4	13	-1.94	0.049	L	-0.53	4	2	74	389	4.16	MTK3	MFK7
22	8	35	4	18	-2.77	0.004	L	-0.64	0	2	92	533	3.94	MTK3	MFK7
23	8	62	11	28	-2.56	0.009	L	-0.44	10	5	72	718	5.18	MTK3	MFK8
24	6	31	17	3	2.91	0.003	R	0.70	2	4	77	521	3.57	MTK3	MFK9
25	13	42	6	21	-2.69	0.006	L	-0.56	9	4	68	626	4.03	MTK3	MFK9
26	9	23	14	1	3.10	<0.001	R	0.87	5	7	56	402	3.43	MTK3	MFK9
27	23	27	4	13	-1.94	0.049	L	-0.53	0	1	94	417	3.88	MTK3	MFK9
28	14	44	14	18	-0.53	0.597	N	-0.13	12	4	67	684	3.86	MTK3	MFK9
29	6	38	6	18	-2.25	0.023	L	-0.50	11	4	62	549	4.15	MTK3	MFK9
30	2	29	1	17	-3.54	<0.001	L	-0.89	1	1	90	407	4.28	MTK3	MFK9

Note z: binomial z test, LI: Lateralization Index, MTP: Mahua-Tree-'Pench' study site, MTK: Mahua-Tree-'Kanha' study site, MFP: Mahua-Flock-'Pench' study site, MFK: Mahua-Flock-'Kanha' study site

Table S2 Individual data on feeding on sacred fig tree, *Ficus religiosa* fruits

Individual	Time from landing to first peck, s	Total fruits ingested	Lateral pecks		z	p	pref	LI	Total feeding time, s	Igestion rate, fruits/ min	Tree	Flock
			R	L								
'Pench' stydy site												
1	17	38	18	0	4.01	<0.001	R	1.00	402	5.67	FTP1	FFP1
2	24	24	11	5	1.25	0.210	N	0.38	307	4.69	FTP1	FFP1
3	4	51	5	24	-3.34	<0.001	L	-0.66	685	4.47	FTP1	FFP1
4	19	27	13	18	-0.72	0.473	N	-0.16	424	3.82	FTP1	FFP1
5	13	52	4	24	-3.59	<0.001	L	-0.71	672	4.64	FTP1	FFP2
6	10	50	14	2	2.75	0.004	R	0.75	587	5.11	FTP1	FFP2
7	20	51	17	3	2.91	0.003	R	0.70	654	4.68	FTP1	FFP3
8	25	57	22	12	1.54	0.121	N	0.29	711	4.81	FTP1	FFP3
9	14	46	23	5	3.21	<0.001	R	0.64	500	5.52	FTP1	FFP3
10	15	18	12	3	2.07	0.035	R	0.60	219	4.93	FTP1	FFP3
11	16	29	3	17	-2.91	0.003	L	-0.70	392	4.44	FTP2	FFP4
12	19	26	14	3	2.43	0.013	R	0.65	318	4.91	FTP2	FFP5
13	14	43	15	0	3.61	<0.001	R	1.00	524	4.92	FTP2	FFP5
14	10	54	2	16	-3.06	0.001	L	-0.78	646	5.02	FTP2	FFP5
15	16	22	13	2	2.58	0.007	R	0.73	272	4.85	FTP2	FFP5
16	5	44	17	6	2.09	0.035	R	0.48	570	4.63	FTP2	FFP5
17	8	24	16	1	3.40	<0.001	R	0.88	285	5.05	FTP3	FFP6
18	14	34	3	16	-2.75	0.004	L	-0.68	442	4.62	FTP3	FFP6
19	17	59	5	15	-2.01	0.041	L	-0.50	703	5.04	FTP3	FFP6
20	6	43	27	8	3.04	0.002	R	0.54	516	5.00	FTP3	FFP6
21	4	63	17	3	2.91	0.003	R	0.70	701	5.39	FTP3	FFP6
22	23	22	4	12	-1.75	0.077	N	-0.50	348	3.79	FTP3	FFP6
23	14	57	19	10	1.49	0.136	N	0.31	720	4.75	FTP3	FFP7
24	16	71	20	6	2.55	0.009	R	0.54	654	6.51	FTP3	FFP7
25	29	22	11	4	1.55	0.118	N	0.47	327	4.04	FTP3	FFP7
Kanha' study site												
1	16	43	2	15	-2.91	0.002	L	-0.76	519	4.97	FTK1	FFK1
2	17	58	26	6	3.36	<0.001	R	0.63	657	5.30	FTK1	FFK1
3	31	64	17	10	1.15	0.248	N	0.26	724	5.30	FTK1	FFK1
4	22	52	12	13	0	1	N	-0.04	699	4.46	FTK1	FFK1
5	3	23	16	0	3.75	<0.001	R	1.00	268	5.15	FTK1	FFK2
6	5	25	3	15	-2.59	0.008	L	-0.67	295	5.08	FTK1	FFK2
7	19	36	15	0	3.61	<0.001	R	1.00	421	5.13	FTK1	FFK3
8	10	53	4	22	-3.33	<0.001	L	-0.69	619	5.14	FTK1	FFK3
9	17	19	16	0	3.75	<0.001	R	1.00	158	7.22	FTK2	FFK4
10	22	33	20	0	4.25	<0.001	R	1.00	323	6.13	FTK2	FFK5
11	17	45	19	5	2.65	0.007	R	0.58	493	5.48	FTK2	FFK5
12	19	41	21	5	2.94	0.002	R	0.62	503	4.89	FTK3	FFK6
13	9	23	14	3	2.43	0.013	R	0.65	274	5.04	FTK3	FFK6
14	16	62	14	5	1.84	0.064	N	0.47	649	5.73	FTK3	FFK6
15	3	55	15	2	2.91	0.002	R	0.76	612	5.39	FTK3	FFK7
16	13	51	29	5	3.94	<0.001	R	0.71	566	5.41	FTK3	FFK7
17	19	39	9	13	0.64	0.523	N	-0.18	541	4.33	FTK3	FFK7
18	13	29	16	3	2.75	0.004	R	0.68	293	5.94	FTK3	FFK7

Note z: binomial z test, LI: Lateralization Index, FTP: Fig-Tree-'Pench' study site, FTK: Fig-Tree-'Kanha' study site, FFP: Fig-Flock-'Pench' study site, FFK: Fig-Flock-'Kanha' study site.

Table S3 Multinomial regression analysis of the impact of the study site ('Pench' and 'Kanha') on the distribution of left (L), right (R) and non-lateralized (N) pigeons

(a) Feeding on mahua flowers

Model Fit Measures

Model	Deviance	AIC	R^2_{McF}
1	150	158	0.002

Model Coefficients - pref

Individual preference	Predictor	Estimate	SE	Z	p
L - R	Intercept	1.099	0.408	2.691	0.007
	Place: 'Kanha' – 'Pench'	-0.336	0.613	-0.549	0.583
N - R	Intercept	0.405	0.456	0.888	0.374
	Place: 'Kanha' – 'Pench'	-0.272	0.690	-0.394	0.694

(b) Feeding on sacred fig fruits

Model Fit Measures

Model	Deviance	AIC	R^2_{McF}
1	84.9	92.9	0.005

Model Coefficients - pref

pref	Predictor	Estimate	SE	Z	p
N - R	Intercept	-0.773	0.494	-1.567	0.117
	Place: Kanha – Pench	-0.238	0.765	-0.312	0.755
L - R	Intercept	-0.773	0.494	-1.567	0.117
	Place: Kanha – Pench	-0.526	0.817	-0.644	0.520

Table S4 Linear regression analysis testing the relationships between lateralization of birds (based on LI) and the particular tree, where the data were collected

(a) Feeding on mahua flowers

Model Fit Measures

Model	R	R^2
1	0.500	0.250

Model Coefficients - LI

Predictor	Estimate	SE	t	p
Intercept [a]	0.070	0.331	0.21	0.833
Flock:				
MFP2 – MFP1	-0.295	0.438	-0.67	0.504
MFP3 – MFP1	-0.400	0.662	-0.60	0.548
MFP4 – MFP1	-0.820	0.524	-1.57	0.123
MFP5 – MFP1	-0.036	0.419	-0.09	0.932
MFP6 – MFP1	-0.530	0.524	-1.01	0.316
MFP7 – MFP1	-0.448	0.406	-1.11	0.274
MFP8 – MFP1	-0.378	0.388	-0.97	0.335
MFP9 – MFP1	-0.575	0.438	-1.31	0.195
MFP10 – MFP1	-0.248	0.406	-0.61	0.543
MFP11 – MFP1	-0.860	0.524	-1.64	0.106
MFP 11 – MFP1	-0.400	0.662	-0.60	0.548
MFK1 – MFP1	0.100	0.468	0.21	0.832
MFK2 – MFP1	0.273	0.468	0.58	0.562
MFK3 – MFP1	0.068	0.438	0.15	0.878
MFK4 – MFP1	-0.765	0.524	-1.46	0.150
MFK5 – MFP1	-1.070	0.662	-1.62	0.112
MFK6 – MFP1	-0.540	0.438	-1.23	0.223
MFK7 – MFP1	-0.424	0.419	-1.01	0.316
MFK8 – MFP1	-0.510	0.662	-0.77	0.445
MFK9 – MFP1	-0.219	0.396	-0.55	0.583

Note [a] Represents reference level. MFP: Mahua-Flock-'Pench' study site, MFK: Mahua-Flock-'Kanha' study site

(b) Feeding on sacred fig fruits

Model Fit Measures

Model	R	R^2
1	0.490	0.240

Model Coefficients - LI

Predictor	Estimate	SE	t	p
Intercept [a]	0.140	0.318	0.44	0.663
Flock:				
FFP2 – FFP1	-0.120	0.550	-0.22	0.829
FFP3 – FFP1	0.418	0.449	0.93	0.361

FFP4 – FFP1	-0.840	0.711	-1.18	0.247
FFP5 – FFP1	0.276	0.426	0.65	0.522
FFP6 – FFP1	-0.067	0.410	-0.16	0.872
FFP7 – FFP1	0.300	0.485	0.62	0.541
FFK1 – FFP1	-0.118	0.449	-0.26	0.796
FFK2 – FFP1	0.025	0.550	0.05	0.964
FFK3 – FFP1	0.015	0.550	0.03	0.978
FFK4 – FFP1	0.860	0.711	1.21	0.236
FFK5 – FFP1	0.650	0.550	1.18	0.247
FFK6 – FFP1	0.440	0.485	0.91	0.372
FFK7 – FFP1	0.353	0.449	0.78	0.439

[a] Represents reference level. FFP: Fig-Flock-'Pench' study site, FFK: Fig-Flock-'Kanha' study site

Table S5 Comparisons of LI scores across different trees at two study sites

**Food object**	**Stydy site**	**KW test statistic**	**p**
Mahua flower	'Pench'	1,27	0,530
	'Kanha'	6,67	*0,036*
Sacred fig fruit	'Pench'	0,14	0,935
	'Kanha'	3,04	0,228

Dunn's multiple comparisons test for KW test on Mahua trees in 'Kanha'

Comparison	Mean rank diff,	Adjusted P Value
MTK1 vs. MTK2	10,96	*0,034*
MTK1 vs. MTK3	6,21	0,279
MTK2 vs. MTK3	-4,75	0,747

Note KW: Kruskal-Wallis, MTK: Mahua-Tree-'Kanha' study site

Table S6 Linear regression analysis testing the relationships between lateralization of birds (based on LI) and the particular feeding flock, the pigeon belonged to.

(a) Feeding on mahua flowers

Model Fit Measures

Model	R	R^2
1	0.494	0.244

Model Coefficients - LI

Predictor	Estimate	SE	t	p
Intercept [a]	0.070	0.329	0.21	0.832
Flock:				
MFP2 – MFP1	-0.295	0.436	-0.68	0.501
MFP3 – MFP1	-0.400	0.659	-0.61	0.546
MFP4 – MFP1	-0.820	0.521	-1.57	0.121
MFP5 – MFP1	-0.036	0.417	-0.09	0.931
MFP6 – MFP1	-0.530	0.521	-1.02	0.313
MFP7 – MFP1	-0.448	0.403	-1.11	0.271
MFP8 – MFP1	-0.378	0.386	-0.98	0.333
MFP9 – MFP1	-0.575	0.436	-1.32	0.192
MFP10 – MFP1	-0.248	0.403	-0.62	0.541
MFP11 – MFP1	-0.707	0.466	-1.52	0.135
MFK1 – MFP1	0.100	0.466	0.21	0.831
MFK2 – MFP1	0.2733	0.466	0.59	0.560
MFK3 – MFP1	0.0675	0.436	0.15	0.877
MFK4 – MFP1	-0.7650	0.521	-1.47	0.148
MFK5 – MFP1	-1.0700	0.659	-1.62	0.110
MFK6 – MFP1	-0.5400	0.436	-1.24	0.221
MFK7 – MFP1	-0.4240	0.417	-1.02	0.313
MFK8 – MFP1	-0.5100	0.659	-0.77	0.442
MFK9 – MFP1	-0.2186	0.394	-0.56	0.581

[a] Represents reference level

(b) Feeding on sacred fig fruits

Model Fit Measures

Model	R	R^2
1	0.490	0.240

Model Coefficients - LI

Predictor	Estimate	SE	t	p
Intercept [a]	0.140	0.318	0.44	0.663
Flock:				

FFP2 – FFP1	-0.120	0.550	-0.22	0.829
FFP3 – FFP1	0.418	0.449	0.93	0.361
FFP4 – FFP1	-0.840	0.711	-1.18	0.247
FFP5 – FFP1	0.276	0.426	0.65	0.522
FFP6 – FFP1	-0.067	0.410	-0.16	0.872
FFP7 – FFP1	0.300	0.485	0.62	0.541
FFK1 – FFP1	-0.118	0.449	-0.26	0.796
FFK2 – FFP1	0.025	0.550	0.05	0.964
FFK3 – FFP1	0.015	0.550	0.03	0.978
FFK4 – FFP1	0.860	0.711	1.21	0.236
FFK5 – FFP1	0.650	0.550	1.18	0.247
FFK6 – FFP1	0.440	0.485	0.91	0.372
FFK7 – FFP1	0.353	0.449	0.78	0.439

[a] Represents reference level. MFP: Mahua-Flock-'Pench' study site, MFK: Mahua-Flock-'Kanha' study site, FFP: Fig-Flock-'Pench' study site, FFK: Fig-Flock-'Kanha' study site.

Table S7 Comparison of LI scores between a real flock and ten simulated flocks, which contain randomly assigned individuals from other flocks to form a synthetic flock (SynFlock) of the same size

Real flock	SynFlock1	SynFlock2	SynFlock3	SynFlock4	SynFlock5	SynFlock6	SynFlock7	SynFlock8	SynFlock9	SynFlock10
Mahua trees in 'Pench' study site										
0.24	0.73	0.73	0.73	0.37	0.37	0.37	-0.89	0.73	-0.89	0.37
-0.56	0.52	-0.76	0.52	0.52	-0.16	-0.50	-0.50	-0.16	-0.50	-0.76
-1.00	-1.00	-0.50	-0.50	-0.50	-0.50	-0.50	-0.50	-1.00	-1.00	-0.50
0.60	-0.46	0.70	0.70	0.70	-0.46	-0.46	0.60	-0.46	-0.04	0.70
-0.43	-0.10	0.67	0.67	0.67	-0.71	-0.87	-0.71	0.67	0.67	-0.10
-0.50	-1.00	0.56	-0.73	-0.85	-1.00	-0.85	-0.85	-0.73	-0.85	-0.85
-0.52	0.73	-0.55	-0.69	-0.55	-0.69	0.73	-0.11	0.20	-0.65	0.20
-0.29	-0.33	-0.58	-1.00	-0.33	-0.58	-0.58	-0.58	-0.33	-0.33	-0.33
Kruskal-Wallis test: H = 6.98, p = 0.728 (Dunn's post test - real flock as a control group: p > 0.05).										
Mahua trees in 'Kanha' study site										
0.70	0.41	0.60	-0.50	0.41	0.60	0.41	0.41	0.60	-0.50	0.60
-0.56	0.27	0.24	0.24	0.52	0.24	0.24	0.52	0.52	0.27	0.52
0.87	-0.68	0.70	1.00	-0.68	-0.47	-0.68	0.70	-0.68	0.70	1.00
-0.53	-0.76	-0.63	-0.63	-0.63	-0.63	-0.76	-0.63	-0.63	-0.63	-0.76
-0.13	-1.00	-1.00	-1.00	-1.00	-1.00	-1.00	-1.00	-1.00	-1.00	-1.00
-0.50	-0.47	-0.31	-0.60	-0.31	-0.31	-0.60	-0.50	-0.50	-0.31	-0.31
-0.89	-0.56	0.60	-0.53	-0.56	-0.56	0.60	0.60	-0.64	-0.64	-0.64
Kruskal-Wallis test: H = 4.30, p = 0.933 (Dunn's post test - real flock as a control group: p > 0.05).										
Fig trees in 'Pench' study site										
0.88	-0.16	1.00	-0.66	1.00	1.00	-0.16	0.38	0.38	1.00	0.38
-0.68	-0.71	-0.71	0.75	-0.71	0.75	-0.71	0.75	0.75	0.75	-0.71
-0.50	0.29	0.29	0.60	0.60	0.29	0.64	0.60	0.29	0.60	0.70
0.54	-0.70	-0.70	-0.70	-0.70	-0.70	-0.70	-0.70	-0.70	-0.70	-0.70
0.70	0.65	1.00	1.00	0.65	-0.78	0.73	0.48	1.00	-0.78	0.65
-0.50	0.54	0.47	0.47	0.54	0.31	0.31	0.31	0.31	0.31	0.31
Kruskal-Wallis test: H = 2.52, p = 0.991 (Dunn's post test - real flock as a control group: p > 0.05).										
Fig trees in 'Kanha' study site										
0.76	-0.04	0.63	0.63	-0.04	-0.76	0.63	0.26	-0.04	-0.76	0.26
0.71	-0.67	1.00	-0.67	-0.67	-0.67	-0.67	1.00	-0.67	1.00	1.00
-0.18	1.00	-0.69	1.00	-0.69	-0.69	1.00	-0.69	-0.69	1.00	1.00
0.68	0.47	0.65	0.62	0.65	0.62	0.47	0.65	0.65	0.62	0.65
Kruskal-Wallis test: H = 10.42, p = 0.405 (Dunn's post test - real flock as a control group: p > 0.05).										
